# Health Effects of Exposure to Natural Arsenic in Groundwater and Coal in China: An Overview of Occurrence

**DOI:** 10.1289/ehp.9268

**Published:** 2007-01-09

**Authors:** Guangqian Yu, Dianjun Sun, Yan Zheng

**Affiliations:** 1 The Center for Endemic Disease Control, Chinese Center for Disease Control and Prevention, Harbin Medical University, Harbin, Heilongjiang Province, People’s Republic of China; 2 State Key Lab of Environmental Geochemistry, Institute of Geochemistry, Chinese Academy of Sciences, Guiyang, Guizhou Province, People’s Republic of China; 3 Lamont-Doherty Earth Observatory of Columbia University, Palisades, New York, USA; 4 Queens College, City University of New York, Flushing, New York, USA

**Keywords:** arsenic, arsenicosis, China, coal, groundwater, health effect

## Abstract

Between 2001 and 2005, 21,155 of 445,638 wells in 20,517 villages in 292 counties in 16 provinces from China, or 5% of wells, were found to contain > 50 μg/L arsenic (As) by field testing with the Merck As kit. We achieved quality assurance of analysis of at least 10% of the wells containing > 50 μg/L As using hydride generation atomic fluorescence spectrometry and silver dithiodicarbomate spectrometry. Our best estimate of the population exposed to > 50 μg/L As in drinking water was 582,769. This is probably an underestimate for China because of the limited area surveyed. In a survey of 135,492 individuals in eight provinces, we used the National Diagnosis Standard for Endemic Arsenicosis and identified 10,096 cases of arsenicosis with various degrees of skin lesions. The arsenicosis occurrence rate of 7.5% is likely an overestimate, because the survey focused more on known and suspected endemic areas of arsenicosis. The occurrence of arsenicosis correlates positively with the percentage of wells containing > 50 μg/L As, or at a ratio of 1 to 5%. Based on both the amount of As in well water and the rate of occurrence of arsenicosis, Shanxi province, Inner Mongolia autonomous region, and Jilin province are the top three areas in China as of 2005 for exposure to endemic As from drinking water. Our survey also identified exposure to high levels of As from wells in several provinces and from the indoor burning of coal containing high levels of As in Shaanxi province. These areas, however, have not had any reports of previous arsenicosis endemics. In the endemic areas, the average rate of occurrence of arsenicosis at advanced stages was 1.2%, possibly because of a long exposure time of > 20 years; the rate of occurrence increased to 2.7% when we included a high dose of As exposure from the indoor burning of coal. Mitigation to reduce As exposure remains a challenge in rural China.

Evidence for elevated arsenic concentrations in groundwater of geologic origin in aquifers of fluvial and deltaic river delta formations has been increasing over the past 20 years worldwide (Smedley and Kinniburgh 2002). Although the calamity of arsenic (As) exposure in Bangladesh may have been the most well-known endemic because of the magnitude of the exposure (British Geological Survey and Department of Public Health Engineering of Bangladesh 2001; Zheng et al. 2004), many other regions in the world, including China, have well-documented As endemics with high occurrence rates of arsenicosis. This documentation has been possible because these regions have longer histories of exposure (Wang and Huang 1994). Consequently, biomedical investigations of the health effects of As began in the 1980s, but these studies were rarely published in English. This represents a loss of opportunities in raising the awareness of the health problems related to As exposure in China and in reducing the exposure that has already led to human suffering. To this end, this mini-monograph, “Occurrence and Health Effects of Arsenic in China,” includes five articles that report the health effects of As in the exposed population and describe the mitigation approach used to reduce As exposure.

Elevated As in groundwater used as drinking water has been reported in the Xinjiang autonomous region (Wang and Huang 1994); the Inner Mongolia autonomous region (Luo et al. 1993; Smedley et al. 2003); Shanxi, Jilin, and Qinghai provinces; and the Ningxia autonomous region (Sun 2004; Wang et al. 2003) in China. Other routes of As exposure are food and air contaminated by As via domestic coal combustion, for example, when chili peppers and corns are dried over open stoves burning coal containing As in Guizhou province (Belkin et al. 1997; Finkelman et al. 1999, 2003; Zheng et al. 1999). The population exposed to As has been difficult to estimate, partly because as the exposure increases over time, so does the population. The total population in the aforementioned regions with endemic arsenicosis has been estimated from approximately 0.5 million (Jin et al. 2003) to as high as approximately 2 million in the mid-1990s (Cao 1996; Peterson et al. 2001), an estimate difficult to confirm. Approximately 200,000 people in Guizhou were reported to reside in the area at risk of exposure to milligram levels of As through the food and air contaminated by the burning of coal (Liu et al. 2002). Perhaps because of the lack of resources in screening As in groundwater in vast regions of northern China and analyzing As and other contaminants in coal-bearing regions in central China, regions with problems of As exposure are often identified only after the health effects of arsenicosis become evident in humans. Sun (2004) pointed out that cases of arsenicosis were identified in northern (Shanxi province and Inner Mongolia autonomous region), northeastern (Jilin province), and northwestern (Ningxia autonomous region, Qinghai province, Xinjiang autonomous region) China, all of which later were found to have elevated levels of As in groundwater.

Are there reasons to be concerned about a geographically wider occurrence of elevated levels of As in groundwater in northern China where groundwater is an important and often the only source of drinking water? Reducing groundwater in Quaternary (defined as the last 2 million years, the most recent geologic period) aquifers with elevated levels of As has also been noted elsewhere in the world, including Taiwan (Tseng et al. 1968), Vietnam (Berg et al. 2001), West Bengal, India (Bhattacharya et al. 1997), and Bangladesh (Nickson et al. 1998; Zheng et al. 2005). A revealing and comprehensive study concluded that hydro-geochemical conditions leading to high concentrations of As in groundwater (up to 1,480 μg/L) in the Quaternary aquifers in Inner Mongolia are similar to those found elsewhere (Smedley et al. 2003). Groundwater wells have been installed into the extensive Quaternary deposits in China to provide drinking water in rural areas [e.g., see Figure 1 in Smedley et al. (2003)]. The As concentrations and the potential for the occurrence of arsenicosis as a result of these wells have not been carefully evaluated.

From 2001 to 2005, the Chinese government implemented a program of arsenic mitigation and management, with joint support from UNICEF (United Nations Children’s Fund). The program began to ascertain the extent and the distribution of As in ground-water through the initial testing of well water for drinking in areas where cases of arsenicosis had been identified or where individuals were suspected of developing arsenicosis. At this stage, such a testing strategy is justified, as those areas that were identified will take priority in receiving alternative drinking water supplies.

In this article, we first report results of the tests performed to determine the level of As in well water. We also report the results of the survey used to identify individuals who had developed arsenicosis. Both the testing and the survey were conducted between 2001 and 2005. We used the results of the survey to estimate the population exposed to elevated levels of As in drinking water in the regions described previously and to assess the rate of occurrence of arsenicosis in order to establish priorities in mitigation. The survey was not used to fulfill the objectives of a cohort study to evaluate the health effects of As.

We then introduce two articles in this devoted to various aspects of the health effects of As exposure in Xinjiang, Shanxi (Wang et al. 2007), and Inner Mongolia (Sun et al. 2007). We also review the impact of As exposure on human health via food and air during the indoor burning of As-containing coal in unventilated stoves in Guizhou. Two articles in this mini-monograph describe the genetic damage in this population (Zhang et al. 2007) and the initial success in mitigation to reduce As exposure (An et al. 2007). Finally, we discuss mitigation plans to reduce As exposure in China from 2006 to 2011.

## Methods

### Water sampling and testing

Water sampling and testing encompassed a total of 445,638 wells in 16 provinces from 2001 to 2005 (Table 1). These wells were located in 20,517 villages in 292 counties in Inner Mongolia and in the provinces of Shanxi, Xinjiang, Ningxia, Jilin, Liaoning, Qinghai, Sichuan, Anhui, Heilongjiang, Henan, Gansu, Jiangsu, Yunnan, and Hunan. The survey took more than 1 year in some of these areas (Ma et al. 2003; Shen et al. 2005).

Areas to be screened were selected on the basis of the following criteria: *a*) the presence of endemic arsenicosis that was confirmed by previous medical examination reports; *b*) the presence of suspected endemic arsenicosis because of the geographic proximity of villages experiencing the same endemic (this included all villages immediately adjacent to any village with endemic arsenicosis); and *c*) occasionally, the presence of suspected endemic arsenicosis based on previous hydrogeologic investigations. The screening strategy for each village was as follows: *a*) All drinking water wells were tested in villages where either high levels of As were present in the drinking water or cases of arsenicosis had been previously reported; also tested were villages bordering these contaminated areas. *b*) Ten percent of the drinking water wells were tested in villages where the As assay had not been performed. Each village was divided into five sections: east, south, west, north, and center. Wells were randomly selected within each section. This 10% sampling method has been shown to be effective in locating wells with high levels of As in a given village, is 95% reliable, and allows a 10-fold increase in the number of villages to be surveyed without increasing the amount of materials and supplies used in the testing process (Sun 2004). Using the Merck arsenic kit imported from Germany (Merck KGaA Merckoquant Arsen Test; Darmstadt, Germany) for onsite testing, a team of medical professionals examined the As concentration in drinking water wells.

If drinking water was found to contain As exceeding 50 μg/L, which is the Chinese maximum contaminant level for As in drinking water [State Environmental Protection Administration (SEPA) 2007], samples were quantified selectively in an environmental testing facility in each county, town, or village using silver dithiodicarbomate spectrometry (DDCAg) or using hydride generation atomic fluorescence spectrometry (HG-AFS) according to the standard As analysis protocol GB/T8538-1995 (Ministry of Health 2007). All testing facilities followed the same quality assurance/quality control (QA/QC) protocol established for the analysis of environmental As in China where GB (GuoBiao) is the National Standard Method (Ministry of Health 2007). The proportion of water samples measured varied among the provinces. HG-AFS was used in three areas (Jiangsu, Shanxi, Henan) to quantify 80–100% of the water samples containing > 50 μg/L As. DDCAg was used in nine areas (Xinjiang, Jilin, Sichuan, Yunnan, Gansu, Inner Mongolia, Anhui, Shandong, Liaoning) to quantify 10–80% of the water samples containing > 50 μg/L As. No laboratory measurements were performed in four provinces (Qinghai, Heilongjiang, Hunan, Ningxia). The program will continue well-water testing from 2006 to 2010 but with an improved sampling strategy and a QA/QC protocol for laboratory measurements that will include wells containing < 50 μg/L As.

### Arsenicosis patient identification protocol

From 2001 to 2003, 135,492 people from eight provinces or autonomous regions were examined according to the protocol described below (Table 2). Not all provinces or autonomous regions conducted or completed a final tally of arsenicosis cases either because of the limited financial resources or the lack of technical expertise in making such a diagnosis.

The National Diagnosis Standard for Endemic Arsenicosis WS/T211-2001 (Sun et al. 2004) was used by trained biomedical personnel from provincial Centers for Endemic Disease Control to identify and categorize the cases of arsenicosis during the survey. This standard protocol was established and recently revised in 2001 by a national committee of specialists in the areas of clinical medicine, pathology, toxicology, and endemic diseases in China before our study was conducted. The standard has been used to diagnose arsenicosis that developed from exposure to As in drinking water and other sources such as food and air from coal combustion. Briefly, palms of the hands, soles of the feet, and parts of the body trunk were examined for symptoms of skin lesions, which included pigmentation, hyperpigmentation (melanosis), hypopigmentation, keratosis, hyperkeratosis, skin ulceration, and skin cancers (Bowen disease), and each category of clinical symptoms was assigned a rank. The arsenicosis patients were then classified “suspected,” “mild,” “moderate,” “severe,” and “skin cancer” with increasing ranking order (Table 2). Biopsies were performed on patients suspected of having skin cancer to confirm a diagnosis. The ethics of the study plan was approved by the same national committee of experts that acted on behalf of the Institutional Review Committees of all the provincial CDCs that carried out the medical examination. The study plan included obtaining informed consent at the time of the examination.

## Results

Because the scope of the surveys described in the mini-monograph is limited, we recognize that the problem of chronic As exposure may be more widespread than we describe below. Ultimately, more extensive surveys are needed to prevent severe health effects, as cluster areas of As endemics in China are usually identified only after severe health outcomes become evident. Because our survey was guided by the known or suspected cases of arsenicosis, our results most likely show a higher occurrence of arsenicosis.

### Distribution of As in well water

**Figure 1:**
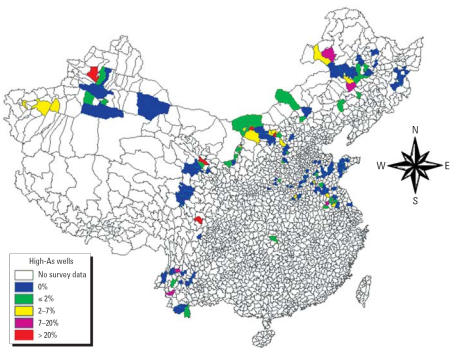
Percentage of wells containing high concentrations of As (> 50 μg/L) at the county level in China as of 2005. Percentages are based on field tests of 445,638 wells.

**Figure 2:**
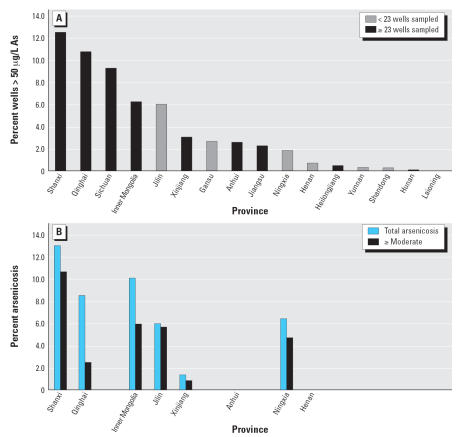
(*A*) Bars indicate the percentage of wells containing high levels of As in 16 provinces where testing was performed. Gray indicates that wells tested in each village from these provinces (*n* = 6) is less than the median value of 23 wells tested per village in all 16 provinces surveyed. Therefore, these provinces were undersampled. (*B*) The occurrence rate of arsenicosis in 8 provinces surveyed. Blue and black bars indicate the percentages of total arsenicosis and the percentage of advanced arsenicosis classified as moderate and greater, respectively.

The highest concentration of As was determined to be > 1,000 μg/L (Table 1). A map showing the percentage of wells with As levels exceeding 50 μg/L for each county also shows that a high percentage (> 7%) of wells with > 50 μg/L As at the county level was found in northeastern (Jilin), northern (Inner Mongolia, Shanxi, Qinghai), northwestern (Xinjiang), and southwestern (Sichuan, Yunnan) China (Figure 1). The number of wells containing high levels of As (> 50 μg/L) was determined to be 21,155 of the total 445,638 drinking water wells surveyed in 20,517 villages in 292 counties in 16 provinces (Table 1). These wells were approximately 5% of the wells surveyed, although the percentages differed significantly among the provinces: 12.5% in Shanxi; 10.8% in Qinghai; 9.3% in Sichuan; 6.3% in Inner Mongolia; 6.1% in Jilin; 3.0% in Xinjiang; 2.7% in Gansu; 2.6% in Anhui; 2.3% in Jiangsu; 1.9% in Ningxia; < 1% in Henan, Heilongjiang, Yunnan, Shandong, and Hunan; and none in Liaoning (Figure 2). In Liaoning, where 3,500 wells were tested in three counties, not a single well containing high levels of As was found (Table 1).

Of 21,155 villages tested, 1,294 had at least one well containing high levels of As. This was approximately 6.3% of the villages tested, which is a higher percentage than that of the wells that were found to contain high levels of As. The higher percentage occcurred because if only one well exceeded 50 μg/L, then that village was classified as a village with high levels of arsenic. On average, 41 ± 41 wells were tested in each village,, but it could be as low as 3 wells per village in Yunnan or as high as 140 wells per village in Liaoning (Table 1). In seven provinces (Jilin, Gansu, Ningxia, Henan, Yunnan, Shandong, Hunan), the number of wells tested was less the median value of 23 wells tested per village (Figure 2), suggesting that sampling density could be improved further in these provinces.

The distribution of wells containing high levels of As is spatially heterogeneous within each province (Figure 1). The percentage of villages with high levels appears to correlate with the percentage of wells also with high As levels for each province, with Xinjiang and Heilongjiang above the trend and Shanxi below the trend. This suggests that in Xinjiang and Heilongjiang, the As-containing wells are located in more villages, whereas in Shanxi the opposite is true. The percentage of counties with high levels of As, however, showed a very weak correlation with the percentage of wells with high levels. This suggests that although not perfect, using the village to estimate population exposed to As is better than using the county (see below). When ranked according to the number of villages where As-containing wells are located, Inner Mongolia, Jilin, Xinjiang, Shanxi, and Ningxia were the top five areas.

### Populations exposed to high levels of As

The population residing in the 21,155 villages tested for As in drinking water was more than 20 million, but the population per village varied widely from region to region, with a minimum of 113 persons in Ningxia to a maximum of 4,558 persons in Shandong (Table 1). The variable population density is another reason for using census data to estimate exposed populations in villages with high As levels. The population residing in 1,294 villages with high levels in 98 counties in 13 provinces (2 provinces did not report data) was 582,769. We consider this the best estimate of people exposed to drinking water containing high levels of As in China as of 2005.

### Occurrence of waterborne arsenicosis

The data set for the occurrence rate of arsenicosis is not nearly as complete as we would have liked. Nevertheless, of 135,492 people examined in 8 provinces, 10,096 cases of arsenicosis were diagnosed, with arsenicosis occurring at the rate of 7.5% (Table 2). The rate of occurrence for arsenicosis at advanced stages, including moderate, severe, and skin cancers, was 1.2%, or 1,716 people (Table 2). The majority of these cases belonged to the suspected or mild categories. When ranked by rate of occurrence, the top five areas with high levels of As were Shanxi, Inner Mongolia, Qinghai, Ningxia, and Jilin (Table 2; Figure 2). Because medical examinations to detect arsenicosis were not conducted, no data was available for Sichuan, where almost 10% of the wells contained high levels of As in the areas tested (Figure 2). The rate of occurrence of arsenicosis in Qinghai is likely to be biased because of a small sample size of 724 villagers from 16 villages with known cases of arsenicosis in the past (Table 2). In Ningxia, the high rate of occurrence was puzzling, given the relatively low percentage of wells containing high levels of As (Figure 2), except we noted that no patients had an advanced degree (severe and skin cancer) of arsenicosis (Table 2).

**Figure 3:**
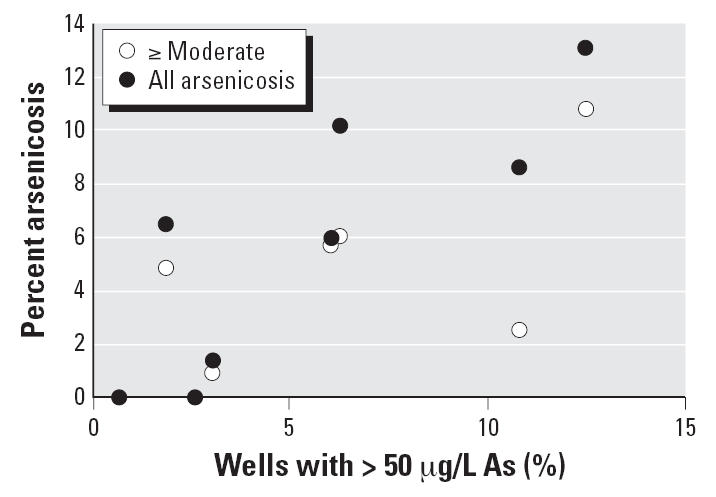
The correlation between the percentage of wells containing high concentrations of As (> 50 μg/L) with the percentage of arsenicosis in each province.

We were not surprised that the percentage of arsenicosis cases correlates positively with the percentage of wells containing high levels of As (Figure 3; *R*^2^ = 0.70). This suggests that on average the concentrations of As to which the population was exposed were not significantly different. This is consistent with the data shown in Table 1. Ningxia, and to a lesser extent Inner Mongolia, showed a higher prevalence of arsenicosis in relation to the the percentage of As-containing wells in these areas (Figures 2, 3). Our data show that the occurrence of arsenicosis should be evaluated in several other provinces, with the priority given to Sichuan, followed by Gansu and Jiangsu.

We have ranked the top five provinces according to the percentages of wells and villages containing high levels As and the percentage of arsenicosis patients. Three provinces were among the top five for all three categories: Shanxi, Inner Mongolia, and Jilin. Qinghai and Ningxia were among the top five in two categories. Sichuan and Xinjiang were among the top five in one category. Sichuan province could well be placed in more categories if data were available on individuals with arsenicosis. Although these rankings are not perfect, our results suggest these areas should be given priority in receiving alternative water supplies.

Several observations discussed below remain puzzling and worthy of future studies. Lower rates of arsenicosis occurrence at or above a moderate degree were found in Jilin (0.8%) and Ningxia (0.6%), despite the high prevalence of arsenicosis (Table 2). We do not know whether this is because of a shorter exposure time, lower concentrations of As in the water, or other factors. In both Ningxia and Jilin, wells containing high levels of As are located over a substantial geographic area. Although data have not been compiled, anecdotally only a few individuals from Anhui, Qinghai, and Sichuan, where As concentrations in individual wells could be hundreds of micrograms per liter, were reported as having severe symptoms of arsenicosis. In these three provinces, wells containing high levels of As were clustered in only a few villages.

## Discussion

### Uncertainty in estimates of the population exposed to high levels of As

Using As data on > 400,000 wells, we estimated that approximately 0.6 million people from 16 provinces in our survey were at risk of exposure to high levels of As in drinking water. Based on As data from 53,805 wells in 21 provinces, Jin et al. (2003) estimated an exposed population of 522,566 and found high levels of As (> 50 μg/L) in villages in 8 provinces: Inner Mongolia, Shanxi, Xinjiang, Jilin, Ningxia, Qinghai, Anhui, and Beijing. Our findings are consistent with Jin et al. (2003). Using As concentration data from 28,000 drinking water samples in 31 provinces, Zhang and Chen (1997) estimated that 3.34 million people were exposed to As at concentrations between 50 and 100 μg/L in drinking water, and that another 2.29 million people were exposed to > 100 μg/L As (Zhang and Chen 1997). This estimate of 5.5 million people is 10 times our current estimate and is probably too high, most likely because too few wells were used to estimate population at risk in too large an area. However, our estimate of approximately 0.6 million people—our best estimate of the population exposed to waterborne As—is likely low because our survey is limited geographically (Figure 1).

### Health outcome of drinking water As exposure

A set of well-conducted and internationally recognized studies based on the population exposed to As in groundwater in Taiwan quantified the risks of skin lesions and cancers (Tseng et al. 1968), various internal cancers (Chen and Lin 1994), and noncarcinogenic effects such as hypertension and Blackfoot disease (Chen et al. 1997). Unfortunately, a significant shortcoming of our study is that our results cannot contribute to a better dose–response relationship of exposure to inorganic As. However, the correlation between the percentage of wells containing high levels of As with the rate of occurrence of arsenicosis (Figure 3) suggests that chronic exposure to 50 μg/L As generates significant public health problems, and in this case, endemics of arsenicosis. Individuals in China with arsenicosis have shown symptoms of various skin lesions such as hyperpigmentation, hypopigmentation, and hyperkeratosis (Wang et al. 1997a, 1997b). Arsenicosis led to skin cancer, internal organ tumors (Zhou et al. 2002), and damage to the nervous(Li et al. 1998), cardiovascular, cerebrovascular, digestive, and genitourinary systems (Su and Jin 2005).

The health outcomes that result from long-term exposure to As in a large population could be used to anticipate future health outcomes for regions such as Bangladesh, where approximately 30% of wells exceeded 50 μg/L As and exposure was relatively recent, especially if the mitigation measures do not rapidly reduce As exposure (van Geen et al. 2002). If the relationship identified in our survey between the occurrence of arsenicosis and the percentage of wells containing high levels of As were the same for the population of Bangladesh, then an alarming rate of arsenicosis, approximately 30%, would be anticipated for Bangladesh a decade or two later.

An exposure time of approximately 20 years also appears to be a key factor in more individuals developing advanced degrees of arsenicosis, for example, moderate and greater, as observed in the As-endemic area in China. An exposure time of approximately 10 years or shorter is sufficient to cause easily recognizable arsenicosis symptoms, especially if the As concentration is > 150 μg/L. The history of how areas with endemic arsenicosis were discovered in China supports this view. For example, for villagers in Shanxi, Inner Mongolia, and Xinjiang, where the time of exposure to high levels of As in drinking water has been either long or the dose has been high, the rates of occurrence of arsenicosis at or greater than a moderate degree were high, with values of 2.4, 1.4, and 0.9%, respectively (Table 2).

#### Xinjiang

The first area of endemic arsenicosis was discovered in Kuitun, Xinjiang autonomy region of China in 1983 (Wang et al. 1997b). In Kuitun, villagers drank river water containing very little As until the late 1960s but switched to drinking As-containing groundwater at the beginning of the 1970s. Wells were sunk to depths as deep as 660 m in thick layers of sediments in the rapidly subsidizing Dzungaria (Junggar) Basin on the north side of the Tianshan Mountains. By the early 1980s, many villagers developed hyperpigmentation and hypopigmentation on chest and abdomen. On palms of the hands and soles of the feet, hyperkeratosis was common. Villagers reported unbearable ache throughout the body and fatigue. Some developed angiocardiopathy or cancer (Wang and Wu 2004). These arsenicosis symptoms were shown to have a dose–response relationship with well water containing As concentrations up to 750 μg/L (Wang et al. 1986). Water from Kuitun and other areas of Xinjiang also contains high levels of fluoride—up to 21.5 mg/L. Note that the maximum permissible level of fluoride in China is 1 mg/L (SEPA 2007). Beginning in the mid-1980s, water containing low levels of As was provided to many of the areas with cases of arsenicosis in the Dzungaria Basin region of northern Xinjiang. However, even after 15 years, this population still had higher mortality rates because of heart disease and malignant tumors despite of improvement of skin lesion symptoms (Wang et al. 1996).

#### Inner Mongolia

Beginning in the early 1990s, cases of arsenicosis emerged in the Hetao Plain area of Inner Mongolia (Gao 1990), including Huhhot Basin (Fan et al. 1993; Luo 1993; Zhang et al. 1994). Use of groundwater in this area began in 1978. In some cases groundwater from different depths was used to reduce fluoride exposure from groundwater containing high levels of fluoride with the unintended consequence of introducing As into the drinking water system (Sun et al. 2007). But mostly, the switch to tube wells in this region is a result of improved economic conditions after the Cultural Revolution, circa 1966–1976. Sun et al. (2007) compared urinary As metabolites in children and adults and found that children had a higher percentage of dimethylarsenic acid (DMA) than adults.

#### Shanxi

The occurrence of arsenicosis was first recognized in Shanxi in 1994 (Cheng et al. 1994), and there again most wells were installed beginning in 1978. Similar to Inner Mongolia, an exposure time of approximately 12–15 years produced notable areas with endemic arsenicosis, mainly in the Datong and Jinzhong basins. In an ecologic study of approximately 700 eight- to twelve-year-old children, Wang et al. (2007) showed that exposure to As lowered children’s IQ. Recent screening for As-containing groundwater continues to identify new areas with high levels of As in drinking water, with the number of cases of arsenicosis probably higher than that reported in Table 1, which was based on survey data obtained in 2001.

### Health outcome of As exposure related to indoor coal usage

Other known routes of As exposure are from food and air contaminated with As via domestic coal combustion, for example, when chili peppers and corns are dried over an open stove burning As-containing coal (An et al. 1992). China is the world’s largest coal producer and consumer, with vast coal mines located in central China. In addition to arsenicosis, many health problems are also attributed to emissions from coal that contains fluorine, selenium, thallium, and most probably mercury and organic compounds (Finkelman et al. 1999). Below, we review the health outcome in an area well known for the occurrence of arsenicosis—Guizhou province—and a recently recognized area of arsenicosis—Shaanxi province. In both provinces, concentrations of As in coal correlated positively with the rates of arsenicosis occurrence, which were higher for males than for females.

About 0.2 million people reside in four counties in Guizhou province where indoor coal combustion has resulted in 2,848 cases of arsenicosis (Liu et al. 2002). The coal used by residents in the endemic areas contains an average of 524 mg/kg As in Guizhou (Zheng et al. 1999). The amount of daily As exposure was as high as 9 mg, with 50–80% from food, 10–20% from air, and 1–5% from water. Environmental and biomedical screenings conducted since the 1990s identified 8,786 households in 32 villages in nine towns in Xingren, Xingyi, Anlong, and Zhijin counties with coal-related As exposure. This is approximately 47,000 people. In this population, the prevalence of arsenicosis was 6.1% but with a substantially higher percentage of advanced arsenicosis (2.7%) than those exposed to As in water in other regions of China. The rates of occurrence of moderate arsenicosis, severe arsenicosis, and skin cancer were 1.9, 0.7, and 0.08%, respectively.

In addition to more severe arsenicosis, a significantly shorter exposure time averaging 3 years can lead to the onset of arsenicosis in this population exposed to As from coal use (Li and An 2005). Arsenicosis patients in Guizhou province reported symptoms of dazzle, limb anesthesia, tinnitus, limb ache, lachrymation, limb ankylosis, stomachache, anorexia, nausea, constipation, diarrhea, nasal discharge, and chest distress (Li and An 2005). Cirrhosis and ascites accounted for most deaths among the cases of arsenicosis (An and Li 2005). The total cancer mortality rate among individuals with arsenicosis was 77.5 per 100,000 per year and was much higher than that of nonarsenicosis individuals at 13.35 per 100,000 per year. Among arsenicosis cases, lung cancer was the most common cause of mortality, followed by liver, skin, stomach, bladder, and rectal cancers (Li et al. 2004). Fortunately, mitigation efforts that took place between 2004 and 2005 have mostly eliminated the source of As exposure through a health education campaign for stove improvement or replacement and the closing of the coal pits that contained high levels of As (An et al. 2007).

Shaanxi province first reported cases of arsenicosis caused by the burning of coal rich in As at the beginning of this millennium (Tang et al. 2002). To date, about 58,256 people from 1,665 villages in 124 towns in eight counties have been exposed to this source of As. In this population, 11,219 cases of arsenicosis were diagnosed and 4,561 were suspected cases. The rate of occurrence of arsenicosis was 19.3%, which is higher than that in Guizhou, with occurrence rates for moderate and severe arsenicosis of 5.2 and 1%, respectively. Skin lesions included hypopigmentation (22.3%), hyperpigmentation (20.5%), and hyperkeratosis (0.75%) (Bai et al. 2006). This recent finding of arsenicosis in Shaanix that results from the use of coal containing high levels of As is troublesome, given the widespread, unregulated coal usage in rural China.

### Mitigation target

The results of our survey indicate the need for continuing the screening process for As in groundwater, together with identifying cases of arsenicosis. From 2006 to 2011, another 12,000 villages will be surveyed. The criteria for choosing these villages are as follows: *a*) target villages with groundwater wells installed into Quaternary sedimentary aquifers in large sedimentary basins where groundwaters are known to be anoxic; and *b*) target villages with known or suspected occurrence of arsenicosis or other skin lesions.

Our results provide the basis for mitigation planning by the Chinese Key Endemic Disease Control Program (2004–2010) to reduce exposure to As. The central government of China has committed 2 billion RMB (principal Chinese trading currency) (US$250 million) for funding alternative water supply and stove improvement projects from 2006 to 2011 in areas using water and burning coal containing high levels of As that have been identified through this study. We have identified 1,294 villages with wells containing high levels of As (Table 1); of those, 388 villages with cases of arsenicosis will be provided a new source of drinking water that will meet all requirements of the national drinking water standards by the end of 2010 (SEPA 2007).

Installation of new ventilated stoves will be completed by 2008 in areas with health problems resulting from the burning of As-containing coal. An et al. (2007) have shown that installation of ventilated stoves must be combined with effective health education to reduce As exposure resulting from coal combustion. Therefore, the mitigation goals are *a*) to achieve a correct operating rate of 95% for the ventilated stove; *b*) to increase awareness to 85 and 75% in students and mothers, respectively, of the harmful health effects of exposure to burning As-containing coal. Monitoring As in coal, food, indoor air, and in urine will be conducted to assess the effectiveness of the stove improvement program.

Arsenic mitigation involves many government agencies and requires collaboration. A first step would be to establish a central database for hydrogeologic, epidemiologic, and water resources data to assist decision making. A China Arsenic Mitigation Network has recently been established, with support from UNICEF, to promote knowledge sharing for better decision making. Better regulations are needed to establish a more consistent framework for handling arsenicosis as well as other endemic diseases. We are also exploring region-specific, local market economic approaches to reduce As exposure, and better methods for increasing awareness of arsenicosis.

## Figures and Tables

**Table 1:** Distribution of wells containing high levels of As in 16 provinces in China.

		Areas tested				> 50 μg/L As					
Province	Geographic location	Wells (*n*)	Villages (*n*)	Counties (*n*)	Population (*n*) of villages	Wells (*n*)	Villages (*n*)	Counties (*n*)	Population exposed (*n*)	Maximum As (μg/L)	Year of sampling
Shanxi^b^	N	48,583	2,120	30	1,455,294	6,077	144	18	115,865	≥ 500	2001–2003
Qinghai^b^	NW	10,003	102	18	106,344	1,081	30	7	21,745	≥ 500	2002–2003
Sichuan	SW	3,870	53	3	36,000	359	8	1	860	≥ 600	2003
Inner Mongolia^b^	N	130,104	2,691	23	1,429,564	8,142	324	18	109,161	≥ 600	2001–2003
Jilin^b^	NE	42,900	2,063	9	1,027,198	2,598	283	6	127,920	≥ 500	2002–2003
Xinjiang^b^	NW	29,747	1,086	12	1,226,090	903	193	12	100,000	≥ 500	2002–2003
Gansu	NW	5,016	538	13	495,059	133	33	2	22,954	≥ 250	2005
Anhui^b^	E	24,033	739	5	115,427	630	45	3	2,833	≥ 1,000	2003
Jiangsu	E	9,604	410	15	773,609	220	No data	3	No data	≥ 300	2005
Ningxia^b^	NW	27,432	2,517	22	285,144	510	112	6	34,590	≥ 100	2002–2003
Henan^b^	N	28,068	1,635	61	1,470,112	192	4	3	7,855	≥ 500	2003
Heilongjiang	NE	43,344	420	15	184,542	220	73	5	No data	≥ 100	2003
Yunnan	SW	9,535	2,978	16	1,029,755	33	10	5	6,839	≥ 200	2005
Shandong	N	19,899	2,419	46	10,782,718	49	30	8	31,799	≥ 50	2003
Hunan	SW	10,000	721	1	614,877	8	5	1	348	≥ 50	2005
Liaoning	NE	3,500	25	3	13,000	0	0	0	0	< 50	2002
Total		445,638	20,517	292	21,044,733	21,155	1,294	98	582,769		2001–2005

Abbreviations: E, east; N, north; NE, northeast; NW, northwest; SW, southwest.

a Provinces are listed in descending order of the percentage of wells with > 50 μg/L As.

b A survey of arsenicosis patients was conducted in these provinces (see Table 2).

**Table 2:** Cases of arsenicosis resulting from exposure to As in drinking water in 8 provinces in China.

	Cases of arsenicosis (*n*)								
Province	Examined	Suspected	Mild	Moderate	Serious	Skin cancer	Total (%)	Percent arsenicosis ≥ moderate	Year of patient identification
Shanxi	31,320	735	2,633	582	156	0	4,106 (13.1)	2.4	2001–2003
Inner Mongolia	48,122	2,110	2,203	582	92	2	4,931 (10.2)	1.4	2001–2003
Qinghai	724	44	18	0	0	0	62 (8.6)	0.0	2003
Ningxia	7,568	127	317	48	0	0	492 (6.5)	0.6	2003
Jilin	2,275	7	111	18	0	0	136 (6.0)	0.8	2001–2003
Xinjiang	24,882	101		236		0	337 (1.4)	0.9	2003
Anhui	16,364	32	0	0	0	0	32 (0.2)	0.0	2003
Henan	4,237	0	0	0	0	0	0 (0.0)	0.0	2003
Total	135,492	3,156	5,282	1,466	248	2	10,096 (7.5)		2001–2003
